# Direct Oral Provocation Test Is Safe and Effective in Diagnosing Beta-Lactam Allergy in Low-Risk Children With Mild Cutaneous Reactions

**DOI:** 10.3389/fphar.2020.01223

**Published:** 2020-08-07

**Authors:** Mara Morelo Rocha Felix, Fábio Chigres Kuschnir

**Affiliations:** ^1^Department of General Medicine, School of Medicine and Surgery, Universidade Federal do Estado do Rio de Janeiro, Rio de Janeiro, Brazil; ^2^Department of Pediatrics, Hospital Federal dos Servidores do Estado, Rio de Janeiro, Brazil; ^3^Department of Pediatrics, Faculty of Medicine, Fundação Técnico Educacional Souza Marques, Rio de Janeiro, Brazil; ^4^Department of Pediatrics, Faculty of Medical Sciences, Rio de Janeiro State University, Rio de Janeiro, Brazil

**Keywords:** drug hypersensitivity, beta-lactams, diagnostic tests, children, skin rash

## Introduction

Beta-lactams (BLs) are frequent causes of drug allergies (Har and Solensky, 2017; Torres et al., 2019). Drug hypersensitivity reactions (DHRs) are defined by the World Allergy Organization (WAO) as “objectively reproducible symptoms or signs initiated by exposure to a defined stimulus at a dose tolerated by normal persons. When immunologic mechanisms have been demonstrated, either antibody or cell-mediated, the reactions should be referred to as drug allergy” (Johansson et al., 2004).

DHRs are generally classified according to the onset time after drug exposure. Immediate reactions occur within the 1^st^ h until 6 h following drug administration and are commonly mediated by IgE (Demoly et al., 2014). Examples include urticaria, angioedema, rhinitis, bronchospasm, or anaphylaxis (Demoly et al., 2014). Non-immediate reactions occur at least 1 h after drug exposure and are mediated by T cells (Demoly et al., 2014). They often appear as a maculopapular exanthema (MPE), but severe reactions may also emerge (Demoly et al., 2014).

About 10% of patients report allergy to penicillin, but 90% or more of these individuals may be able to tolerate penicillins (Har and Solensky, 2017). The misdiagnosis of penicillin allergy results from overestimated reports by health professionals and patients. Some symptoms (e.g., abdominal pain, nausea, headache) are usually side effects, but can be mistaken for allergic reactions (Har and Solensky, 2017). In addition, MPE may have been caused by an underlying infection or even by an interaction between a virus and the antibiotic (Har and Solensky, 2017). Another consideration is that there is a natural decrease in IgE antibodies against a penicillin over time (Har and Solensky, 2017). When assessing individuals with a clear history of immediate reaction and negative allergic tests, we must consider the time elapsed since the subject’s last drug exposure.

The diagnosis of BL allergy begins with clinical history. Some tests, as *in vivo* (skin tests and drug provocation tests) and *in vitro* tests (specific IgE levels, basophil activation tests, lymphocytic transformation tests) may help elucidate the diagnosis (Demoly et al., 2014). A detailed history is crucial for evaluation of BL allergy (Macy, 2014). Nevertheless, it can be vague or imprecise, leading to an incorrect diagnosis (Har and Solensky, 2017). Skin tests (STs) are painful and have suboptimal sensitivity (Moral and Caubet, 2017). The oral provocation test (OPT) is considered the most accurate test, with high positive and negative predictive values (PPV/NPV) (Moral and Caubet, 2017). Thereby, direct OPT without previous STs has been increasingly used in patients, especially children with a history of mild non-immediate reactions to BLs (Moral and Caubet, 2017; Graham et al., 2018; Torres et al., 2019).

Our aim was to review literature on diagnosis of allergy to BLs and to discuss the safety and efficacy of direct OPT in the diagnosis of BL allergy in children.

## Why Is It Important to Correctly Diagnose BL Allergy?

The misdiagnosis of BL allergy may affect the health system in two ways: 1) false allergy label, with an unrealistic increase in incidence of BL allergy and impact on treatment options; and 2) false label of non-allergic, with important consequences in patient safety through prescription errors, leading to more severe reactions (Mayorga et al., 2019).

The patient with “penicillin allergy” is at increased risk of receiving broad-spectrum antibiotics, such as fluoroquinolones and clindamycin, which are associated with an increased prevalence of infections by multi-resistant bacteria (Lee et al., 2000; Macy and Contreras, 2014; van Dijk et al., 2016). In addition to antimicrobial resistance, studies have shown that patients “allergic to penicillin” have a higher frequency of postoperative complications, longer hospital stays, higher treatment costs and a higher rate of treatment failure (Solensky, 2014; Jefferson et al., 2018; Lucas et al., 2019).

## Who Should Be Evaluated for BL Allergy?

Due to the deleterious consequences of a false label of BL allergy, we should evaluate all individuals with a history suggestive of hypersensitivity to BLs (Torres et al., 2019). If the history is incompatible with an allergic reaction, for example, gastrointestinal symptoms, headache, dizziness, or other manifestations suggestive of side effects, the patient can receive treatment with BLs again (Har and Solensky, 2017). On the other hand, if the patient does not know details about his previous reaction, the best approach is to perform a complete investigation with *in vitro* tests, STs (immediate/delayed reading) and OPT. It is important to emphasize that patients who have a family history of allergy to BLs, with no personal history of previous reaction, do not need to be tested and can receive BLs safely (Har and Solensky, 2017).

## How Is the Diagnosis of BL Hypersensitivity Performed Today?

Currently, the diagnosis of BL hypersensitivity is conducted by history, *in vitro* tests, STs and OPTs (Demoly et al., 2014; Har and Solensky, 2017; Torres et al., 2019). *In vitro* tests have low sensitivity, being performed in selected cases (Mayorga et al., 2016). If the patient had an immediate reaction, *in vitro* tests and STs (prick and intradermal tests) with immediate reading are performed. If they are negative, OPT is conducted (Blanca et al., 2009). In the case of mild non-immediate reactions in children, STs are less used and OPT is a safe procedure (Moral and Caubet, 2017; Graham et al., 2018). Mild non-immediate reactions include delayed-appearing urticaria and mild/moderate MPEs. OPT is formally contraindicated if there is history of severe cutaneous adverse reactions (SCARs) such as Drug Reaction with Eosinophilia and Systemic Symptoms (DRESS), Stevens-Johnson syndrome (SJS), and toxic epidermal necrolysis (TEN) (Blanca et al., 2009; Macy, 2014).

The role of STs in mild non-immediate reactions to BLs has been questioned. The accuracy of STs suffers influence of various factors such as pre-test probability and type of reagents used. In Brazil, for example, the major and some of minor benzylpenicillin determinants are not commercially available, which reduces the sensitivity of STs. Recently, a systematic review and meta-analysis assessing the accuracy of STs and specific IgE in evaluation of patients who report allergy to BLs have been published (Sousa-Pinto et al., 2020). They included 105 primary studies (31,761 participants). STs had sensitivity of 30.7% and specificity of 96.8%. Specific IgE had sensitivity of 19.3% and specificity of 97.4% (Sousa-Pinto et al., 2020). Their results suggest that STs (at least in mild non-immediate reactions) and specific IgE have high specificity and NPV, but low sensitivity and PPV (Sousa-Pinto et al., 2020). In immediate reactions, it is difficult to define PPV in individuals with positive STs, as OPT is contraindicated in these patients. In non-immediate reactions, few studies have investigated PPV. Caubet et al. performed STs and OPTs in 88 children with non-immediate reactions to BLs and found a PPV of 36% (Caubet et al., 2011).

## How Can We Improve the Diagnosis of Penicillin Allergy?

To simplify the algorithm and reduce the cost of diagnostic procedures, various investigators are performing direct OPTs without prior STs in low-risk patients. Whether there is agreement on the high-risk patient, there is no uniform definition of low-risk patient. The last EAACI position paper on diagnosis of BL hypersensitivity presented a risk stratification according to the index reaction (Romano et al., 2019). In summary, the high-risk group included those with history of severe immediate reactions (e. g., anaphylaxis, hypotension, laryngeal edema, bronchospasm, urticaria/angioedema) and severe non-immediate reactions (e. g., SJS, TEN, DRESS, serum-sickness-like disease, organ-specific manifestations) (Romano et al., 2019). Mild and moderate MPEs were considered of low risk, especially in children. Mild MPE was defined as “a more or less widespread rash, with less than a week of duration, without systemic involvement” and moderate MPE, those with more than a week of duration, without systemic symptoms (Romano et al., 2019).

First studies evaluating direct OPTs were done with children who presented non-immediate reactions, such as MPE. More recently, children with non-severe immediate reactions and adults were also investigated. Currently, there are already some studies suggesting that this strategy may be safe and effective for a selected group of “low-risk” patients (**Table 1**).

**Table 1:** Studies that evaluated efficacy and safety of direct beta-lactam provocation tests.

**Study**	**Age**	**Country**	**Index reaction**	**Exclusion criteria**	**Diagnostic tests**	**Results**	**Safety**
Vezir et al., 2016	Children and adolescents; 0-18 yo (n=119)	Turkey	Mild non-immediate reactions without systemic involvement (MPE or delayed-appearing urticaria/ angioedema)	Severe reactions (SJS, TEN, DRESS, AGEP, nephritis, pneumonitis, hepatitis, and vasculitis)	Direct OPT (5 doses were administered with 30-min intervals in increasing doses). OPT was continued for 5 days.	OPTs with the suspected drug were performed in 119 patients. Four patients (3.4%) had a reaction during OPT.	No severe reactions were recorded during OPT.
Mill et al., 2016	Children; median age 1.7 yo (n=818)	Canada	History of allergy to amoxicillin (immediate and non-immediate reactions)	SJS/TEN	Direct OPT (10% of the dose of amoxicillin, then 20 minutes later 90% of the dose).	Among all, 770 (94.1%) tolerated the OPT; 17 (2.1%) had immediate reactions and 31 (3.8%) non-immediate reactions.	No severe reactions were recorded during OPT.
Iammatteo et al., 2019	Children (≥ 7 yo) and adults (n=155)	US	History of non-life-threatening reactions to penicillin	Bronchospasm or laryngeal edema requiring intubation; anaphylactic shock; or severe non-IgE-mediated reactions (e. g. SJS, TEN, DRESS, nephritis, hepatitis, anemia, vasculitis, SSLR, pneumonitis). Pregnancy and antihistamine use.	Direct OPT (placebo followed by a 2-step OPT to amoxicillin).	Of 155 patients who completed OPT, 120 (77.4%) had negative tests, 31 (20%) had non-allergic reactions and four participants (2.6%) experienced mild allergic reactions.	No severe reactions were recorded during OPT.
Mustafa et al., 2019	Children and adults (n=363)	US	History of penicillin allergy. For the randomized study, patients needed to be aged 5 to 17 years with a history of cutaneous reaction (> 1 year ago) or aged 18 years and older with a history of cutaneous reaction (> 10 years ago).	Pregnancy. History of a severe cutaneous non IgE-mediated adverse drug reaction or SSLR.	Randomized trial comparing penicillin ST followed by OPT with amoxicillin versus direct OPT. Reagents used for STs were benzylpenicilloyl polylysine (Pre-Pen^®^) and penicillin G 10,000 U/mL.	13 children (< 5 yo) underwent direct OPT (all negative); 159 patients were randomized to direct OPT or ST. STs were negative in 70 of 80 (87.5%) patients. All 70 patients had negative OPTs. Direct OPT was negative in 76 of 79 (96.2%) patients.	No severe reactions were recorded during OPT.
Kuruvilla et al., 2019	Adults (n=50)	US	History of benign rash or benign somatic symptoms, or another unknown history associated with their last penicillin exposure if it occurred > 12 months ago.	Recent reaction (<12 months), a history of a penicillin-associated blistering rash, hemolytic anemia, or organ involvement. Antihistamines or steroids use.	OPT with a single dose of oral amoxicillin 500 mg was performed.	Four patients (8%) were de-labeled based on history. Twenty subjects (40%) were submitted to OPT, and none developed immediate, or delayed hypersensitivity reactions.	No severe reactions were recorded during OPT.

Acute generalized exanthematous pustulosis (AGEP), drug reaction with eosinophilia and systemic symptoms (DRESS); MPE, maculopapular exanthema; OPT, oral provocation test; SJS, Stevens-Johnson syndrome; SSLR, serum sickness-like reaction; ST, skin test; TEN, toxic epidermal necrolysis; US, United States.

Vezir et al. evaluated 119 children with history of mild non-immediate cutaneous reactions induced by BL through direct OPTs (Vezir et al., 2016). Only four (3.4%) reacted with urticaria during OPTs, and there was no severe reaction (Vezir et al., 2016).

In Canada, Mill and colleagues investigated 818 children with suspect allergy (immediate and non-immediate) to amoxicillin (Mill et al., 2016). Exclusion criteria were SJS and TEN. There were no reactions in 770 (94.1%), mild immediate reactions in 17 (2.1%), and non-immediate reactions in 31 (3.8%) patients (Mill et al., 2016). Immediate reactions consisted of hives and non-immediate reactions varied from MPE to serum sickness-like reaction (Mill et al., 2016). They also conducted a follow-up of 250 participants. Of these, 55 received full treatment with amoxicillin, and 49 (89.1%) tolerated the treatment (Mill et al., 2016).

In United States (US), Iammatteo and colleagues investigated patients ≥ 7 years-old with history of mild penicillin allergy through direct OPT with no prior STs (Mill et al., 2016). Of 155 patients who completed OPTs, 120 (77.4%) had negative tests, 31 (20%) had non-allergic reactions and four participants (2.6%) experienced allergic reactions; all were mild (Mill et al., 2016).

In addition, also in the US, Mustafa et al. investigated children and adults with history of penicillin allergy and developed a randomized trial comparing two strategies: ST plus OPT versus direct OPT to amoxicillin in low-risk patients (Mustafa et al., 2019). Of the total, 13 children (<5 years) were submitted to direct OPTs (all were negative) and 159 patients (≥5 years old) with mild cutaneous reactions underwent ST or direct OPT. Eighty patients underwent STs and 70 (87.5%) had negative results. These 70 patients with negative STs underwent OPT (all were negative). Direct OPT was performed in 79 patients and, in 76 (96.2%) participants, they were negative. There was no severe reaction.

Lastly, Kuruvilla et al. evaluated direct OPT without prior STs in adults with a “low-risk” history of penicillin allergy (Kuruvilla et al., 2019). There were 50 patients with penicillin allergy label and 38 of them met their criteria for direct OPT. Four subjects were de-labeled based on history. Twenty participants were submitted to OPT, and none developed hypersensitivity reactions (Kuruvilla et al., 2019).

## Discussion

Evidence is increasingly supporting direct OPT with no prior STs in mild non-immediate reactions to BLs, especially for the pediatric age group. Therefore, many guidelines are already suggesting this approach in the management of BL hypersensitivity in children (Mirakian et al., 2015; Gomes et al., 2016; Romano et al., 2019; Torres et al., 2019). There are some peculiarities in the management of DHRs in children (Gomes et al., 2016). Viral and bacterial infections are important differential diagnoses, as they may act as cofactors or triggers of exanthemas in young children (Gomes et al., 2016). In addition, diagnostic procedures such as intradermal STs are painful and less tolerated in this age group (Gomes et al., 2016). Another aspect is that lifelong avoidance of BLs in children is more difficult (Gomes et al., 2016). Thus, there are more studies investigating direct OPTs in children and adolescents with history of mild non-immediate reactions compared to adults, and their results suggest that this approach is safe and effective in this age group.

Optimization of diagnostic protocols is of outmost importance in penicillin allergy de-labeling programs. This optimization should balance accuracy, risks, costs, and labor-intensity. Direct OPT has many advantages such as being less time-consuming, less expensive and more accurate. To ensure safety, it is essential to perform risk stratification, as OPT can trigger a potentially serious hypersensitivity reaction (Romano et al., 2019). However, what constitutes a “low-risk” BL allergy history is highly variable. Recently, a multicenter Australian study investigated criteria to determine an optimal low-risk definition for penicillin testing (Stevenson et al., 2020). They evaluated 447 patients and found that 97.1% of 244 patients defined as low risk tolerated a direct OPT (Stevenson et al., 2020). They concluded that a history of penicillin-associated exanthema (without angioedema, mucosal ulceration, or systemic involvement), more than 1 year ago, was sufficient to select a patient for a direct OPT (Stevenson et al., 2020).

Several factors should be considered in risk stratification: type of manifestation (exanthema, urticaria, angioedema, and others), chronology (immediate vs. non-immediate), systemic involvement, comorbidities, pregnancy, and time elapsed since the last reaction. The “high risk” patient group generally includes recent reactions (< 1 year ago), patients with comorbidities, pregnant women, systemic involvement (encompassing all SCARs) and immediate reactions with angioedema or anaphylaxis. As highlighted in the previously reported studies, the evaluation of direct OPT was performed excluding high-risk patients (Mill et al., 2016; Vezir et al., 2016; Iammatteo et al., 2019; Kuruvilla et al., 2019; Mustafa et al., 2019).

In summary, BL allergy label is a major public health issue, as it leads to the use of non-BL antibiotics, which may be inappropriate and cause more side effects. On the other hand, traditional diagnostic procedures include many steps that can be an obstacle to the correct diagnosis. Thus, new strategies have been developed to improve the investigation of BL hypersensitivity. Direct OPTs without previous STs in low-risk patients can be a feasible and cost-effective approach in the coming years. This strategy seems to be safe and effective for children with mild non-immediate reactions. However, there are still controversies about which patients should undergo ST versus direct OPT. Further studies, including various populations and age groups, are needed to enable a stronger recommendation in this regard.

## Author Contributions

MF designed, drafted, reviewed and submitted the full document. FK designed and reviewed the full document.

## Conflict of Interest

The authors declare that the research was conducted in the absence of any commercial or financial relationships that could be construed as a potential conflict of interest.
